# Beta Cell Stress to Epitope Diversification: Post‐Translationally Modified Antigens in Type 1 Diabetes

**DOI:** 10.1111/imr.70183

**Published:** 2026-10-07

**Authors:** Megan L. Proffer, Saptarshi Roy, Jon D. Piganelli

**Affiliations:** ^1^ Department of Microbiology and Immunology Indiana University School of Medicine Indianapolis Indiana USA; ^2^ Department of Endocrinology Indiana University School of Medicine Indianapolis Indiana USA; ^3^ Center for Diabetes and Metabolic Diseases Indiana University School of Medicine Indianapolis Indiana USA

**Keywords:** autoimmunity, beta cell stress, epitope spreading, neoepitope, post‐translational modification, Type 1 diabetes

## Abstract

Pancreatic beta cells are active participants in the loss of immune tolerance because their high secretory demand creates exceptional vulnerability to inflammatory exposure, endoplasmic reticulum (ER) stress, calcium dysregulation, and redox imbalance. These conditions can promote enzymatic and nonenzymatic changes in self‐proteins, generating post‐translationally modified (PTM) antigens and neoepitopes that were absent or poorly represented during immune tolerance. This review integrates the contributions with the broader type 1 diabetes (T1D) field to examine how beta cell stress, transglutaminase‐2 dependent reactions, peptidyl‐arginine deiminase mediated citrullination, oxidative modifications, and post‐translational peptide fusion reshape the antigenic landscape. We consider how these products alter antigen presentation and recognition by autoreactive T cells and how an evolving neoepitope repertoire may promote antigenic diversification and epitope spreading. Importantly, clonal recruitment of new determinants must be distinguished from apparent broadening caused by T cell receptor (TCR) cross‐reactivity, dual‐TCR expression, bystander activation, or tissue redistribution. Defining when modified epitopes arise, which are naturally presented, and which responses contribute to pathogenesis could improve biomarker development, patient stratification, and therapeutic strategies designed to reduce beta cell stress, thus enhancing survival and identity maintenance, while restoring immune tolerance.

## 1 Introduction

Elucidating how autoreactive recognition progressively broadens is essential for understanding the progression of chronic immune‐related diseases [1, 2]. In T1D patients, the heterogeneous genetic susceptibility to environmental exposures and pancreatic beta cell stress converge to disrupt immune tolerance and promote CD4+ and CD8+ T cell responses against insulin‐producing cells [3, 4, 5, 6]. Therefore, disease progression is not determined solely by the immune response that initiates islet inflammation but also depends on how continued beta cell injury changes the antigens available for recognition and recruits or expands additional autoreactive responses.

One of the most important features that progresses autoreactive immune recognition is the diversification of an immune response from an initiating determinant to additional epitopes located either within the same protein or across distinct proteins derived from the same target tissue [1, 7]. Intramolecular epitope spreading occurs when recognition expands to new determinants within the same protein, whereas intermolecular epitope spreading involves determinants derived from different proteins within the same tissue or antigenic complex [7]. Classical epitope spreading generally implies the recruitment or expansion of lymphocyte clones with additional antigen specificities [8]. It should be distinguished from TCR cross‐reactivity, in which one receptor recognizes multiple structurally related peptide‐bound major histocompatibility complexes (MHC), from dual‐TCR expression, bystander activation, or tissue redistribution [8, 9]. Each of these processes can create the appearance of a broadened immune response without demonstrating the antigen‐driven recruitment and expansion of a distinct T cell population recognizing a new epitope [9].

In the healthy physiological immune response, limited antigen availability and epitope spreading enable the host to strengthen protective immunity by broadening antigen recognition as pathogens replicate and cause tissue injury which exposes additional determinants [9, 10, 11]. However, pathogen expansion is usually constrained because pathogen clearance progressively removes the antigen source and inflammatory signals. In autoimmunity, however, the target tissue cannot be eliminated without continuing to replenish the self‐antigens that sustain the response. Thus, epitope spreading may consequently become self‐perpetuating rather than self‐limited [1, 10].

This distinction is particularly important in T1D since autoreactive lymphocytes release native proteins, sequestered material, and poorly displayed determinants from injured beta cells [3]. APCs acquire these antigens in an inflammatory environment in which cytokine exposure promotes altered proteolysis, ER stress, and redox imbalances that can impact antigen processing and structure [12, 13, 14]. Presentation of the resulting determinants can recruit additional autoreactive clones, or expand pre‐existing populations, and this intensifies islet inflammation and beta cell stress. The combined tissue injury, antigen availability, APC presentation, and autoreactive recognition can therefore operate as a feed‐forward circuit [15]. This cycle is illustrated in Figure 1.

**FIGURE 1:**
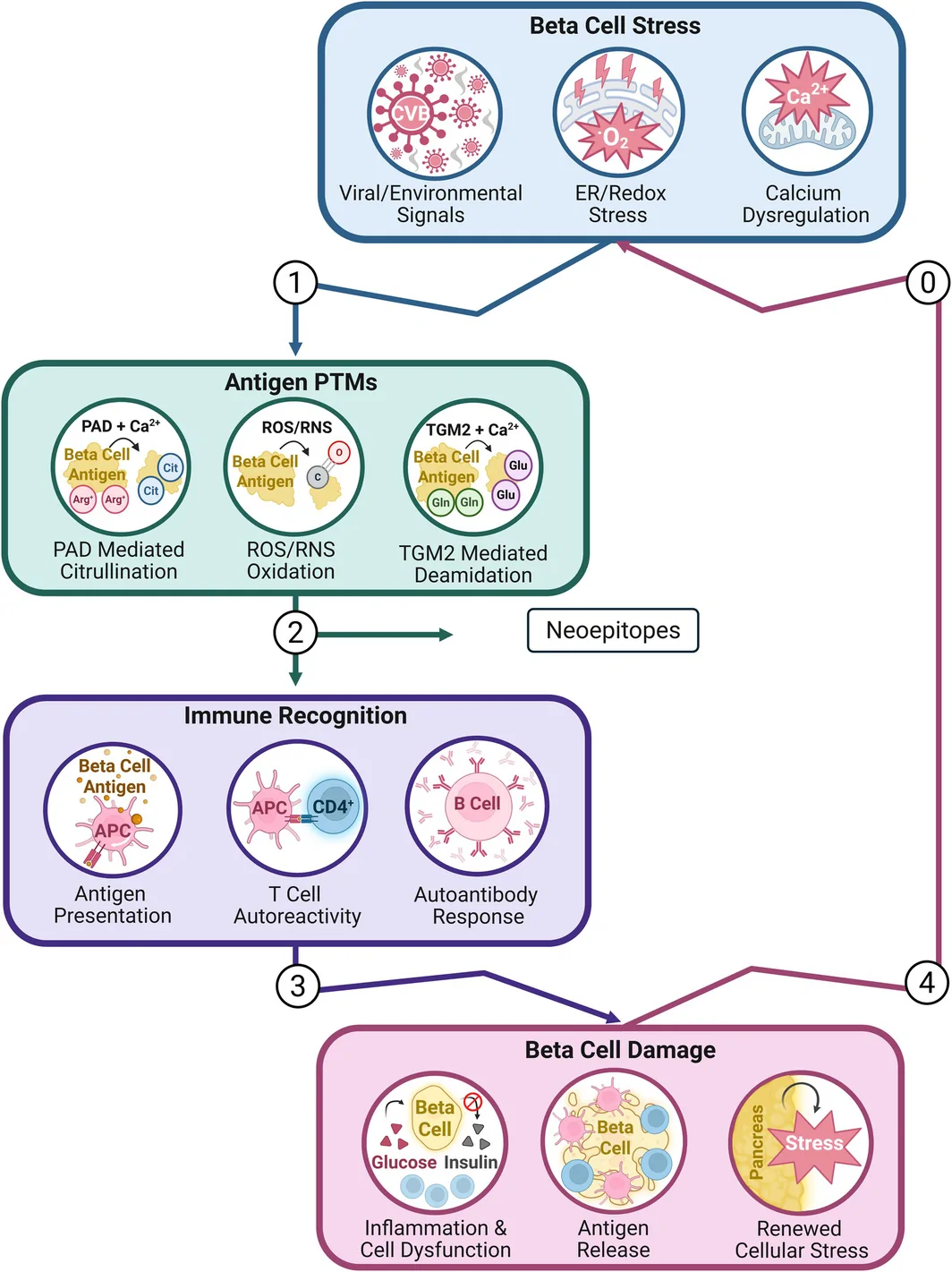
Dynamic beta cell antigenicity. Stress‐induced PTMs create neoepitopes that activate autoreactive lymphocytes, promote beta cell injury, and sustain a feed‐forward cycle of stress, antigen release, and immune recognition. Abbreviations: Antigen‐presenting cell (APC), endoplasmic reticulum (ER), major histocompatibility complex (MHC), peptidyl‐arginine deiminase (PAD), post‐translational modification (PTM), transglutaminase 2 (TGM2).

The emergence of stress‐dependent antigens also exposes an important limitation of immune tolerance when comparing antigen availability during the standard immunological response to autoimmune disease pathways [12, 13]. Central tolerance is established largely against the self‐antigens and peptide structures available to developing lymphocytes in the thymus. Stressed beta cells can subsequently generate peptide combinations or processing byproducts that were absent or poorly represented in the tolerizing repertoire [16]. This concept does not imply that every stress‐associated antigen is pathogenic but establishes a mechanistic route to describe how changes within the target tissue can introduce a previously untolerized antigenic space [12, 13, 15].

Several related terms must be separated when describing this changing antigen landscape. A PTM is a covalent chemical alteration occurring after protein synthesis that can affect protein folding, proteolysis, MHC binding, or receptor recognition [15]. A neoepitope is an antigenic determinant newly created or substantially altered relative to the physiologic antigen repertoire and therefore not effectively represented during tolerance [12, 13]. However, some antigens that were tolerized contain cryptic epitopes, which have a sequence already present in the native protein but are ordinarily processed or presented inefficiently [16].

In the context of T1D, hybrid insulin peptides (HIPs) represent an important class of neoepitopes formed post‐translationally when a proteolytically generated insulin‐ or proinsulin‐derived fragment becomes covalently joined through a new peptide bond to another peptide fragment derived from insulin or a beta cell secretory‐granule protein [17, 18]. This fusion creates a novel, non‐genomically encoded junction that can be presented by MHC molecules and recognized by autoreactive T cells [19, 20]. Although HIPs are commonly classified as PTM neoepitopes, their formation is mechanistically distinct from conventional sidechain PTMs, such as citrullination, deamidation, or oxidation, which chemically alter specific amino acid residues without fusing two peptide sequences [12, 13].

Genetic susceptibility also determines which components of this changing antigen repertoire are displayed to T cells. In NOD mice, I‐A^g7^ is functionally analogous to the major human T1D‐risk molecule HLA‐DQ8; shared peptide‐binding features can support unconventional registers and weakly displayed self‐peptide–MHC complexes [21, 22, 23, 24, 25, 26]. Consequently, autoreactive T cell clonotypes may escape negative selection and later respond when the relevant native or modified peptide–MHC complexes become more abundant or stably presented in the islet [25, 26].

In T1D, HIPs are post‐translational neoepitopes formed when a proteolytically generated insulin‐ or proinsulin‐derived fragment is covalently joined through a new peptide bond to another insulin fragment or beta cell secretory‐granule peptide [12]. This non‐genomically encoded fusion junction can be presented by MHC molecules and recognized by autoreactive T cells. Although HIPs are often grouped with PTM neoepitopes, their formation is mechanistically distinct from conventional sidechain PTMs like citrullination, deamidation, and oxidation that can chemically alter residues without fusing two peptide sequences [17, 18, 19, 20].

This means HIP formation must also be distinguished from altered antigen processing that involves changes in proteolytic cleavage, peptide trimming, or intracellular processing to generate peptides with different boundaries but do not create a novel fusion junction [12, 13, 19, 20]. Alternative translation products represent a distinct source of antigenic novelty and arise during protein synthesis through noncanonical initiation, alternative reading‐frame usage, rapidly degraded defective ribosomal product (DRiP) proteins important for immune system surveillance, or translational errors [27]. However, these mechanisms occur at different stages and generate structurally distinct products, but they can converge functionally by producing peptide–MHC complexes that were absent or poorly represented during thymic selection or peripheral tolerance, thereby creating new opportunities for autoreactive T cell recognition [12, 13].

The beta cell antigenic repertoire therefore includes native targets such as insulin, chromogranin A (ChgA), and islet amyloid polypeptide (IAPP), as well as stress‐dependent variants, HIPs, and alternatively generated products that may alter self‐recognition [19, 21, 28]. These products are particularly relevant because autoreactive T cells may encounter them in inflamed islets even when the corresponding peptide–MHC complexes were scarce during tolerance [22, 23]. This review therefore integrates the broader T1D literature on PTM mechanisms, MHC‐TCR recognition, epitope diversification, biomarkers, and therapy, with emphasis on how these antigens arise, whether they are naturally presented, and whether they recruit new clonotypes or engage cross‐reactive receptors [3].

## 2 The Beta Cell as a Stress‐Sensitive Generator of Neoantigens

Pancreatic beta cells are not passive targets of autoimmunity per se; instead, their normal physiology predisposes this cell to a landscape of opportunities for stress‐related injury. The same physiology that enables them to sense glucose and rapidly produce, process, store, and secrete insulin also places persistent pressure on protein‐folding and quality‐control systems [29]. Consequently, beta cells can alter the antigenic material encountered by the immune system before extensive cell death occurs [6]. Tolerance is established against self‐antigens available during lymphocyte development and maintained through peripheral regulatory mechanisms [13]. Antigenic structures generated or enriched only within stressed beta cells may be absent, rare, or presented differently during immune selection [13, 14]. Once presented in a susceptible inflammatory and MHC context, these stress‐associated antigens can recruit or activate autoreactive lymphocytes, intensify tissue injury, and increase the release and presentation of additional beta cell determinants [13, 14]. Accordingly, beta cell stress can actively reshape the antigenic substrate on which autoimmune recognition operates.

### 2.1 Secretory Burden, Proinsulin Folding, and Constitutive ER Stress

Insulin production places an unusually high biosynthetic demand on the ER in the beta cell. In response to changes in blood glucose, beta cells must rapidly adjust proinsulin synthesis while maintaining correct disulfide‐bond formation, protein folding, and trafficking through the ER and Golgi apparatus [29]. Adaptive UPR pathways elevate chaperone expression, transiently reduce translation, and enhance ER‐associated degradation to preserve beta‐cell function during metabolic stress. The beta cell therefore operates with a degree of constitutive ER stress that is closely coupled to its normal secretory function [13]. The unfolded protein response (UPR), coordinated through IRE1, PERK, and ATF6, initially protects beta cells by reducing protein influx, increasing chaperone capacity, and promoting the disposal of misfolded material [29]. When secretory demand is prolonged or quality‐control mechanisms become insufficient, then an adaptive UPR can shift toward chronic dysfunction and cell death.

Sustained ER stress can change which proteins are retained, degraded, cleaved, or transported into secretory granules, thereby modifying the pool of peptides available for immune recognition [13]. This relationship is especially relevant because several major beta cell autoantigens are concentrated within the insulin secretory pathway [28, 30, 31]. Insulin and proinsulin are processed and stored together with IAPP, ChgA, and other granule proteins [25, 26]. Their high local concentrations and shared proteolytic environment create opportunities for incomplete processing, altered peptide boundaries, and peptide fusion [17, 18, 19]. ER stress is important not only because it can promote beta cell death, but also because it can change the molecular identity of the beta cell before destruction occurs [32].

### 2.2 Environmental and Inflammatory Amplification

The basal stress associated with insulin production can be amplified by environmental and inflammatory signals implicated in T1D [32]. Cytokines released by infiltrating immune cells, including IL‐1β, IFN‐γ, TNF, and Type I interferons, can increase ER and mitochondrial stress, disrupt beta cell identity, alter proteasomal and lysosomal processing, and enhance antigen‐presentation machinery [6, 13, 14, 33, 34]. These effects increase both the generation of unusual antigenic products and the likelihood that beta cell‐derived peptides will be displayed or transferred to APCs. Viral infection or sensing of viral nucleic acids may provide an additional layer of stress. Pattern‐recognition and antiviral interferon signaling can alter protein synthesis while increasing inflammation and MHC expression. Some viral exposures may also disturb ER membranes, mitochondrial function, or intracellular calcium handling [32, 33, 34, 35]. Antiviral responses may remodel the beta cell antigen‐processing environment even when direct cytolysis is limited.

Enteroviruses provide the strongest human viral evidence, although association does not establish that infection initiates T1D. Enteroviral VP1 and low‐level viral RNA have been detected in insulin‐containing islets from subsets of donors with recent‐onset disease [33, 34]. In parallel, viral sensing and Type I interferon signaling can induce HLA Class I hyperexpression, ER‐stress programs, inflammatory chemokines, and beta cell apoptosis [35]. Thus, viral exposure may amplify PTM generation and antigen presentation through interferon‐driven stress even when infection is focal, persistent, or noncytolytic; the magnitude, timing, and causal contribution probably vary among patients [36].

Redox imbalance further connects inflammatory exposure to altered antigen structure. Glucose‐stimulated insulin secretion depends on mitochondrial electron transport to generate ATP, and this metabolism also produces reactive oxygen species (ROS) that are normally detoxified [37, 38]. During high metabolic activity and islet inflammation, increased ROS and reactive nitrogen species (RNS) can overwhelm the comparatively limited antioxidant capacity of beta cells. Excess ROS/RNS can damage proteins and organelles, disrupt disulfide‐bond formation, and generate oxidative or nitrosative modifications that alter protein stability, cleavage, or immune recognition [37, 38].

ER function also depends on tightly regulated luminal calcium. ER calcium depletion impairs calcium‐dependent chaperones and disrupts proinsulin folding, while increased cytosolic calcium can activate stress‐responsive modifying enzymes [39, 40]. Cytokines, viral responses, oxidative injury, and prolonged secretory demand can each disturb calcium homeostasis [32]. These pathways therefore function as an interacting stress network that favors abnormal protein processing and modification before overt beta cell loss [13]. The interacting stress pathways are summarized in Figure 2.

**FIGURE 2:**
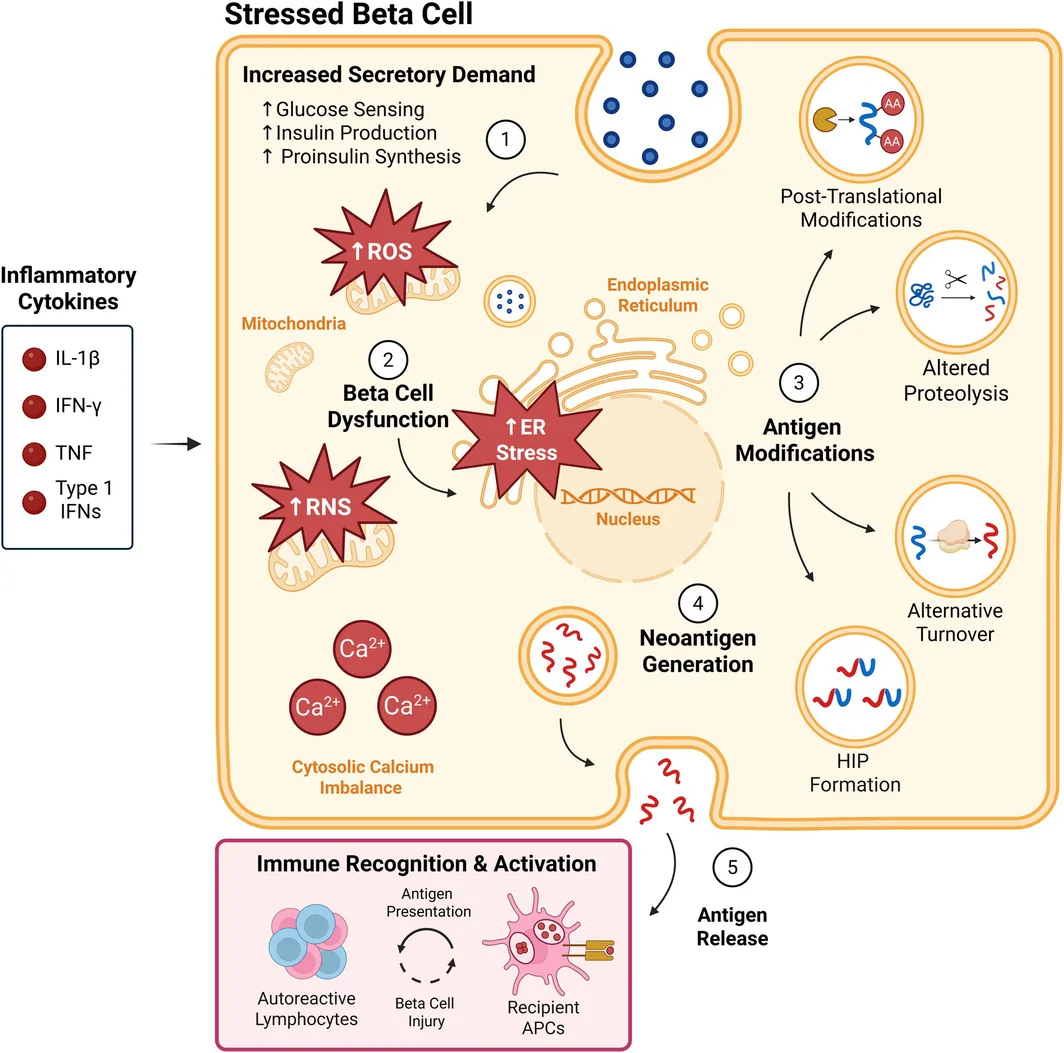
Beta cell stress promotes neoantigen generation and immune activation in type 1 diabetes. Increased secretory demand and inflammatory stress promote ER stress, oxidative/nitrosative stress, mitochondrial dysfunction, and Ca^2+^ dysregulation in beta cells. These changes alter protein modification and processing, generating PTMs, hybrid insulin peptides (HIPs), and other noncanonical antigens. Release and presentation of these antigens can activate autoreactive lymphocytes and further amplify beta cell injury.

### 2.3 From Cellular Stress to Antigen Release and Immune Recognition

Cellular stress can diversify the beta cell antigen repertoire through several distinct routes. Chemical modification can alter amino acid side chains, whereas changes in proteolysis can reveal cryptic determinants or generate peptides with new boundaries. However, separate reactions within dense secretory granules can covalently join peptide fragments to form HIPs [12, 17, 19]. Stress may also affect alternative translation, protein turnover, and the compartments in which antigens are processed [27]. Although these mechanisms produce different molecular products, each can generate peptide–MHC complexes that were absent or poorly represented during thymic selection and peripheral tolerance [13].

Stressed and dying beta cells can release antigenic material through granule turnover, abnormal secretion, extracellular vesicles, apoptotic bodies, and loss of membrane integrity [14]. Local APCs can acquire this material and present it within the islet or pancreatic lymph nodes [41]. Inflammatory signaling simultaneously increases APC activation, creating conditions in which previously ignored determinants can recruit new autoreactive clones or stimulate cross‐reactive receptors [6, 13]. The resulting responses promote additional cytokine production and beta cell injury, further increasing cellular stress and antigen availability. Beta cell dysfunction, neoantigen generation, antigen release, and immune recognition can therefore form a self‐amplifying circuit that supports antigenic diversification during T1D progression.

This framework positions the beta cell as an active determinant of the autoimmune repertoire rather than merely the site of immune attack. Its secretory burden establishes the underlying vulnerability; environmental and inflammatory signals amplify ER, redox, and calcium stress; and altered processing or release determines which antigenic structures become visible to the immune system [13, 29, 32]. The following sections examine the PTM‐generating pathways and modified beta cell antigens that emerge within this environment while distinguishing direct evidence from proposed mechanisms.

## 3 Development of the Stress‐Induced Neoepitope Model in T1D

A central contribution to understanding T1D immunopathology has been the development of a tissue‐centered model in which pancreatic beta cell stress changes the antigenic repertoire encountered by the immune system [15, 29]. Rather than treating beta cells as passive sources of fixed autoantigens, this work proposed and then experimentally tested the idea that secretory stress, inflammatory exposure, calcium dysregulation, and redox imbalance can alter beta cell proteins before terminal cell death [32, 39, 40, 42]. Across conceptual reviews, mechanistic murine studies, human translational experiments, and collaborative investigations, the program helped connect beta cell physiology to PTMs, neoepitope formation, and immune recognition [13]. Table 1 summarizes the conceptual and experimental contributions discussed below.

**TABLE 1:** Contributions to PTM biology in T1D. Conceptual and experimental advances connecting beta cell stress, antigen modification, and autoimmune recognition.

Year	Context	Mechanism	Evidence	Contribution
2015	Beta cell secretory and ER stress	PTMs and modified beta cell epitopes	Conceptual reviews	Stress‐induced antigenic remodeling [15, 29]
2016	ER‐stressed murine islets and insulinoma cells	Ca^2+^‐TGM2; ChgA/WE14‐related antigens	Enhanced diabetogenic CD4^+^ recognition; inhibition by Ca^2+^ chelation or TGM2 suppression	Mechanistic stress‐TGM2 to immunogenicity link [39]
2017	Environmental, inflammatory, and metabolic stress	TGM2, PAD2, and oxidative PTMs	Conceptual review	Unified stress‐to‐neoantigen model [32]
2018	ER‐stressed human islets and beta cell lines	Deamidated IA‐2; citrullinated IAPP	HLA‐DQ8 binding; PTM‐selective human CD4^+^ responses	Human translation of the stress‐PTM pathway [40]
2018	Cytokine‐stressed human islets	Citrullinated GRP78	LC–MS/MS; HLA‐DR4 tetramers; T cell and autoantibody responses	Defined stress‐induced human neoantigen [42]
2021	Beta cell and immune cell crosstalk	PTMs and noncanonical antigens	Conceptual review	Dynamic antigen‐repertoire model [12]
2024	Beta cell and immune system interplay	Cytokine signaling, autophagy, and beta cell stress	Conceptual review	Bidirectional beta cell to immune pathogenesis model [6]

### 3.1 Establishing the Stress‐Induced Neoepitope Model

By 2015, the central gap was no longer simply the identification of beta cell autoantigens or autoreactive T cells but understanding how beta cell physiology and stress could alter antigenic targets and reshape immune recognition [15, 29]. There was evidence that enzymatic and nonenzymatic modifications can change peptide binding to susceptible MHC molecules or alter the surface recognized by a TCR [15]. In parallel, other literature emphasized that the unusually high secretory demand of beta cells produces constitutive ER stress and creates a setting in which abnormal protein folding, processing, and modification may occur [29]. Together, these reviews shifted the investigations from finding native beta cell proteins that are autoantigens to understanding how the beta cell state generates antigenic structures that were absent or poorly represented during immune tolerance.

This framework positioned ER stress as more than a terminal pathway to beta cell death, raising the possibility that changes in beta cell physiology could precede immune‐mediated destruction and alter the antigen repertoire encountered by the immune system [29]. Importantly, PTMs are not necessarily abnormal or intrinsically immunogenic; many occur physiologically, and their recognition is ordinarily constrained by central and peripheral tolerance [15, 29]. These conceptual studies therefore did not establish that stressed beta cells simply generate inherently immunogenic PTMs. Instead, they highlighted the unresolved question of how stress‐dependent changes in antigen abundance, processing, and presentation interact with the peptide‐binding properties of disease‐associated MHC Class II molecules to expose otherwise tolerated PTM‐derived peptides to autoreactive T cells.

This model was subsequently broadened by integrating viral, inflammatory, chemical, metabolic, and oxidative stressors with beta cell ER stress and neoantigen formation [32]. This synthesis proposed a threshold concept: physiological secretory stress may establish basal vulnerability, whereas environmental or inflammatory stress can intensify calcium release and modify enzyme activity sufficiently to increase beta cell immunogenicity [13, 29, 32]. Nevertheless, it remained necessary to show experimentally that ER stress caused modifying‐enzyme activity and that this activity contributed to autoreactive recognition.

### 3.2 Stress‐Dependent Immunogenicity and TGM2 Involvement

By 2016, several investigators directly addressed that mechanistic gap. Earlier studies had shown that synthetic beta cell peptides could become more stimulatory after modification, but they had not established why or where those modifications would arise in intact beta cells [39]. One of these studies tested whether ER stress could increase the immunogenicity of endogenous beta cell antigens and whether calcium‐dependent tissue transglutaminase 2 (TGM2; referred to as Tgase2 in the original study) contributed to that effect. Chemical induction of ER stress with thapsigargin increased unfolded‐protein‐response signaling in primary murine islets without requiring overt apoptosis. When these stressed islets were processed and presented by APCs, they elicited stronger IFN‐γ responses from diabetogenic BDC2.5 CD4+ T cells than unstressed islets [39].

ER stress also increased cytosolic calcium and TGM2 activity. Reducing cytosolic calcium or TGM2 expression diminished the increase in T cell recognition, supporting a functional pathway from ER stress through calcium‐dependent enzyme activity to beta cell immunogenicity. Complementary experiments showed that chemical stress in vitro and physiological stress in vivo could increase ER stress, TGM2 activity, and recognition of insulinoma cells that were otherwise poorly antigenic [39]. These findings were particularly relevant to the ChgA‐related antigen system recognized by BDC2.5 T cells. The WE14 peptide is a naturally occurring ChgA cleavage product and a relatively weak agonist in its unmodified form but becomes substantially more stimulatory following treatment with TGM2 [43, 44].

TGM2 can catalyze deamidation of glutamine residues as well as transamidation or crosslinking reactions, and these outcomes should be described specifically rather than grouped under the imprecise term transglutamination [39]. The same work established that TGM2 is active within stressed beta cells and is required for full stress‐dependent immunogenicity in the experimental system. The causal conclusion should remain appropriately bounded. The study also demonstrated that induced ER stress increased calcium‐dependent TGM2 activity and that TGM2 contributed to enhanced diabetogenic T cell recognition in murine islet and insulinoma models [39]. The contribution was not proof that this pathway independently initiates human disease, but evidence of a causal link between stress, enzyme activity, and immunogenicity.

### 3.3 Extending the Mechanism to Human T1D

The next field gap was translational, even if ER stress generated immunogenic PTM products in murine beta cells, as it remained unclear whether comparable pathways operate in human cells and whether people with T1D carried T cells capable of recognizing the resulting epitopes. A study combining HLA‐DQ8 binding analysis, human T cell cloning, stressed human islets, and a human insulinoma model identified modified epitopes derived from IA‐2 and IAPP that were preferentially recognized in their modified forms [40]. Deamidation of selected IA‐2 glutamine residues and citrullination of IAPP residues affected HLA‐DQ8 binding, TCR recognition, or both [40]. These residue‐specific effects demonstrated that PTMs do not increase immunogenicity through a single mechanism, but that a modification may improve peptide–MHC stability, create a new TCR contact, or combine both effects.

The CD4+ T cell clones isolated from individuals with T1D had proliferated and produced predominantly Th1‐associated cytokines in response to modified peptides, whereas the corresponding native sequences generally produced weaker or undetectable responses. T cells recognizing these epitopes were identified in peripheral blood and in pancreatic‐draining lymph nodes from donors with established T1D, placing PTM‐reactive populations both systemically and near the affected tissue [40]. Stressed primary human islets and βLox5 insulinoma cells also stimulated T cell clones specific for the modified epitopes. Mechanistically, ER stress increased cytosolic calcium and the activities of both TGM2 and peptidyl‐arginine deiminase 2 (PAD2), extending the calcium‐dependent modifying‐enzyme model from murine to human beta cell systems [15, 40]. The calcium‐dependent TGM2 and PAD2 pathways are illustrated in Figure 3.

**FIGURE 3:**
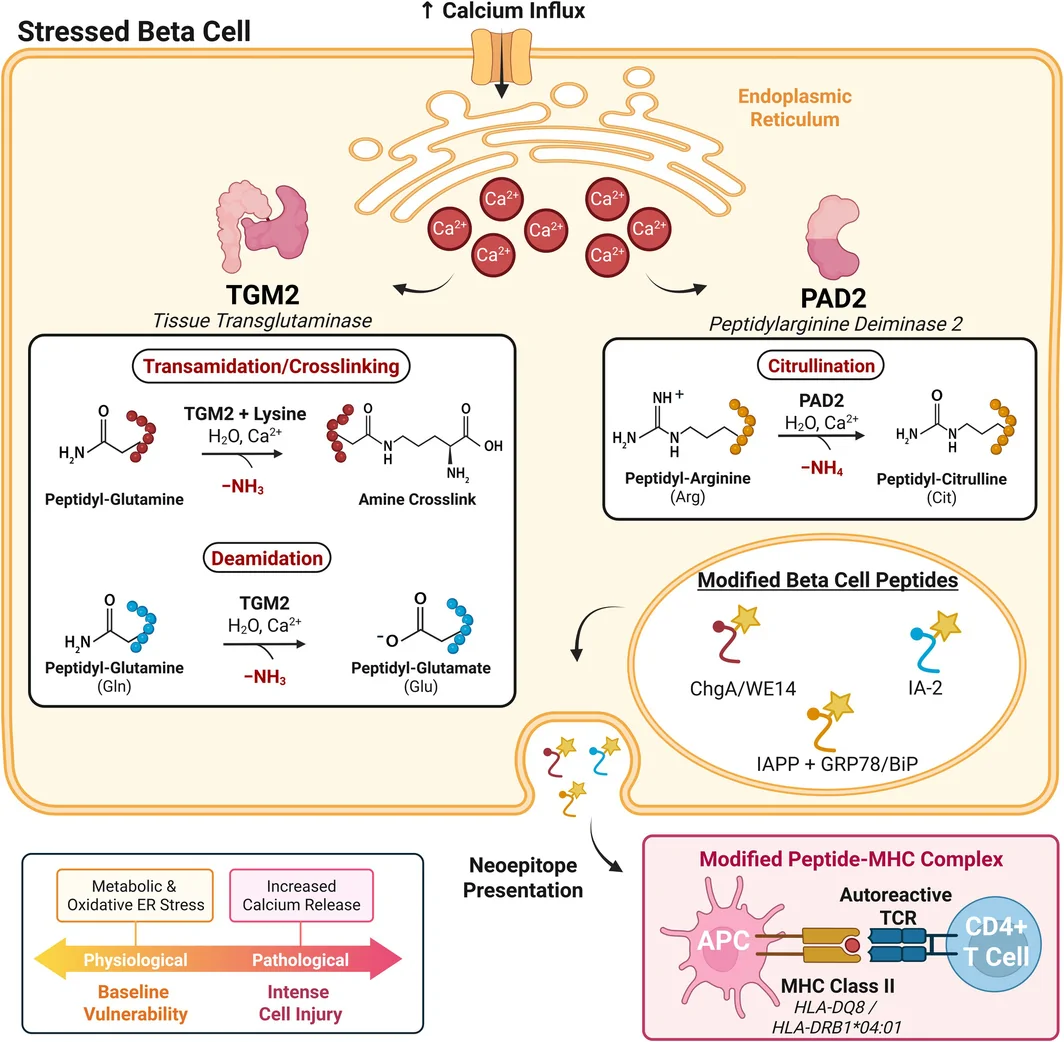
Stress‐induced TGM2 and PAD2 activity generates modified beta cell antigens. Ca^2+^ dysregulation during beta cell stress activates TGM2 and PAD2. TGM2 catalyzes glutamine deamidation (Gln → Glu) and transamidation/crosslinking, whereas PAD2 catalyzes arginine citrullination (Arg → Cit). Modified beta cell peptides can be presented by HLA Class II molecules and recognized by autoreactive CD4^+^ T cells.

Activation of modification‐selective T cell clones by stressed islet preparations strongly supports natural generation of the relevant antigens, but it does not by itself identify the modified residue within the naturally presented peptide–MHC complex. Moreover, detecting PTM‐reactive T cells in established disease cannot determine whether response‐initiated autoimmunity arose during epitope spreading, or expanded secondarily after tissue injury [40]. The work nevertheless established human relevance by connecting beta cell stress, modifying‐enzyme activity, HLA‐restricted recognition, and modification‐selective CD4+ T cell responses [15].

In 2018, collaborative work provided complementary evidence for citrullination. Exposure of human islets to IL‐1β, TNF‐α, and IFN‐γ induced conversion of arginine 510 within glucose‐regulated protein 78 (GRP78) to citrulline, which was confirmed by mass spectrometry. A citrullinated GRP78 epitope was presented by HLA‐DRB1*04:01 and elicited increased CD4+ T cell and autoantibody responses in individuals with T1D relative to controls [42]. This study strengthened the evidence that inflammatory stress and ER‐to‐cytosol calcium flux can activate calcium‐dependent modifying enzymes and generate a defined human beta cell antigen engaging both cellular and humoral immunity [40, 42].

### 3.4 Synthesis: A Feed‐Forward Model

The progression of this research supported an integrated, feed‐forward model of beta cell stress and autoimmune diversification. Inflammatory cytokines, viral responses, metabolic demand, chemical exposures, and oxidative injury can intensify the ER and redox stress already associated with beta cell secretory physiology [13, 32, 37, 38]. ER calcium release can increase the activity of TGM2 and PAD enzymes, while ROS/RNS can generate nonenzymatic oxidative modifications [13, 29, 40, 42]. In parallel, stress can alter proteolysis, RNA processing, translation, and secretory‐granule biology, producing several mechanistically distinct classes of neoepitopes.

When these modified or newly processed peptides reach antigen‐presentation pathways, disease‐associated MHC molecules can display structures that were absent or poorly represented during thymic selection [12, 15]. PTMs may increase MHC binding, change TCR‐facing residues, or both [40]. Autoreactive T and B cells that recognize these structures can then amplify islet inflammation and beta cell injury. Additional stress, antigen release, and APC uptake create further opportunities for recruitment of new clonotypes or expansion of existing and cross‐reactive populations [13, 32]. In this model, beta cell stress and immune attack are reciprocal processes rather than a simple linear sequence.

The evidence supporting individual links is not equivalent. Experimentally demonstrated links include ER stress‐dependent calcium release, increased TGM2 and PAD activity, enhanced recognition of stressed murine and human beta cell preparations, cytokine‐induced citrullination of GRP78, and human T cell or autoantibody recognition of defined modified antigens [39, 40, 42]. Associations supporting the model include the presence of PTM‐reactive T cells in blood and pancreatic‐draining lymph nodes, the appearance of carbonylated proteins before hyperglycemia in NOD mice, and recognition of modified antigens in human T1D [37]. Still hypothetical are the complete longitudinal order of these events in individual patients, the degree to which each PTM initiates rather than amplifies disease, and whether PTM formation directly drives recruitment‐based epitope spreading rather than cross‐reactivity or expansion of pre‐existing clones.

## 4 PTMs And Neoepitope Mechanisms Across the T1D Field

PTMs and other forms of noncanonical antigen generation expand the beta cell antigenic repertoire through biochemically distinct mechanisms. These distinct routes to antigen diversification are summarized in Figure 4 and should be evaluated with an explicit hierarchy of evidence. Detection of a modified protein establishes that chemical events can occur in beta cells, but not that its peptides are naturally displayed by MHC molecules [12, 13]. Increased peptide–MHC binding indicates a presentation advantage, whereas ex vivo T or B cell reactivity demonstrates immune recognition without necessarily proving pathogenicity [15, 16]. In vivo beta cell injury, appearance before overt disease, and validation in human tissues all additionally provide noninterchangeable levels of support [12, 13, 15, 16].

**FIGURE 4:**
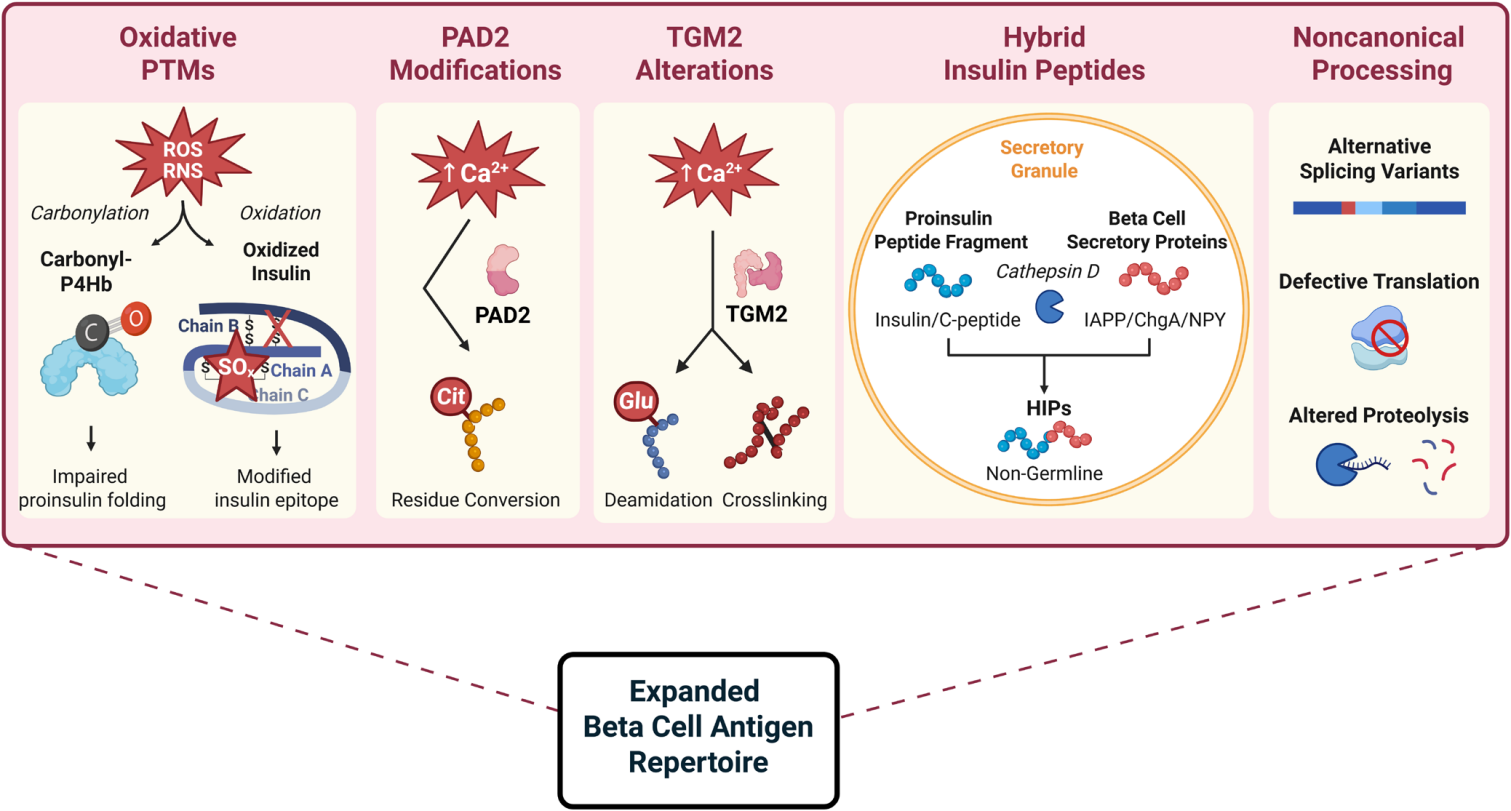
Mechanisms that expand the beta cell antigen repertoire during type 1 diabetes progression. Beta cell stress can diversify self‐antigens through oxidative and carbonyl PTMs, PAD2‐mediated citrullination, TGM2‐dependent deamidation or crosslinking, hybrid insulin peptide formation, and noncanonical processing pathways. These mechanisms generate structurally altered or novel peptides that expand the beta cell antigen repertoire and may promote autoreactive immune recognition.

### 4.1 Oxidative and Carbonyl PTMs as Parallel Stress‐Dependent Pathways

The beta cell is particularly susceptible to oxidative and nitrosative injury because glucose metabolism, disulfide‐bond formation, and inflammatory signaling generate reactive oxygen and nitrogen species, whereas beta cells have limited antioxidant capacity. These species can modify amino‐acid side chains or generate reactive carbonyl intermediates. Oxidation and carbonylation, S‐nitrosylation of cysteine thiols, and nitration of susceptible tyrosines may disrupt protein folding and function, alter proteolysis or peptide–MHC binding, and create new surfaces for immune recognition [38].

Carbonylated protein disulfide isomerase A1, also designated the prolyl 4‐hydroxylase beta subunit (P4Hb), provides an example spanning several levels of evidence. Increased islet protein carbonylation was detected before hyperglycemia in NOD mice, and carbonylated P4Hb was enriched in stressed islets [37, 42]. The modification was associated with impaired glucose‐stimulated insulin secretion and altered proinsulin‐to‐insulin handling, while carbonyl‐P4Hb elicited autoantibody and CD4+ T cell responses in murine and human T1D samples [37, 42]. These findings connect a chemically defined stress product with beta cell dysfunction, temporal appearance before diabetes in a model, and human immune recognition, but do not prove that carbonyl‐P4Hb initiates human T1D or establish its contribution relative to other antigens [37].

Oxidatively modified insulin provides complementary human evidence. Antibodies to oxidized insulin and oxidized insulin‐derived peptides have been detected in T1D and have added information to conventional islet‐autoantibody profiles in longitudinal cohorts; oxidized insulin peptides can also elicit T cell responses [38, 45]. However, antibody reactivity does not demonstrate natural presentation within the islet, and biomarker performance should not be conflated with pathogenic function. Evidence for nitrosylated or nitrated beta cell antigens is less complete: for many targets, the sequence from in situ modification to MHC presentation and disease‐driving recognition remains unproven.

### 4.2 PAD‐Dependent Citrullination

Citrullination is catalyzed by PAD enzymes, which convert the positively charged guanidinium group of peptidyl arginine to the neutral ureido group of citrulline [40, 42]. This reaction is not simply a marker of protein damage but shows that loss of positive charge can change protein conformation, protease cleavage, peptide solubility, MHC binding, and the surface presented to a TCR. PAD enzymes are calcium dependent, making calcium dysregulation during ER and inflammatory stress a plausible link between beta cell state and citrullinated‐antigen formation. GRP78/BiP is among the best‐characterized citrullinated beta cell targets [40, 42, 46, 47, 48]. Inflammatory cytokines increased GRP78 citrullination in human islets, and mass spectrometry identified citrullination at Arg510 [40, 42]. Citrullinated GRP78‐derived peptides were presented in an HLA‐DR4 context and recognized by T cells and autoantibodies from individuals with T1D [42]. The timing of citrullinated GRP78 appearance in preclinical human disease, the abundance of naturally presented citrullinated peptide‐HLA complexes, and the extent to which these responses independently drive beta cell injury remain unresolved. Additional targets show that citrullination is not restricted to one ER chaperone. ER stress‐dependent PAD activity has been associated with generation of citrullinated IAPP epitopes recognized by CD4+ T cells from individuals with T1D, and inflammatory citrullination of glucokinase has been linked to altered enzyme function and autoreactive responses [31, 33, 40, 49].

### 4.3 TGM2‐Dependent Deamidation and Transamidation

It is important to note that deamidation must be distinguished from deamination. In peptide deamidation, an amide‐containing side chain, most commonly glutamine, is converted to a carboxylate‐containing residue, producing glutamate and introducing a negative charge [15]. Deamination is a broader term for the removal of an amino group and should not be used as a synonym. Tissue TGM2 can catalyze glutamine deamidation when water acts as the acyl acceptor. It can also catalyze transamidation, in which a glutamine side chain is cross‐linked to a primary amine, including the ε‐amino group of lysine. These possible products should be specified rather than collectively described as transglutamination [39, 40].

TGM2‐mediated deamidation is particularly relevant to T1D‐associated HLA‐DQ molecules because conversion of glutamine to glutamate may improve occupancy of peptide‐binding pockets that favor acidic residues. Experimental deamidation of islet‐autoantigen peptides has increased binding to HLA‐DQ8 or related susceptible HLA‐DQ molecules and, for selected peptides, enhanced inflammatory T cell recognition [46]. Human islets and dendritic cells can generate deamidated islet antigens under inflammatory conditions, linking tissue modification and antigen processing [47]. It was identified that this ER stress‐elicited TGM2 activity was connected to recognition of deamidated IA‐2 epitopes by CD4+ T cells from people with T1D [31, 40].

The effect is antigen and sequence dependent. Deamidation did not increase the immunogenicity of tested C‐peptide epitopes in individuals with T1D, whereas other deamidated peptides formed in pancreatic islets are recognized by human CD4+ T cells [46, 48]. Each candidate then requires definition of the modified residue, biochemical route, HLA restriction, and naturally processed product. An in vitro gain in HLA binding is insufficient without evidence that the peptide arises in relevant tissue and engages disease‐associated responses.

### 4.4 HIPs As Post‐Translational Fusion Neoepitopes

HIPs are post‐translational neoepitopes generated by covalent fusion of an insulin or proinsulin fragment to a second peptide fragment, often derived from another beta cell secretory‐granule protein such as IAPP or ChgA [17, 18]. Their defining feature is a novel peptide‐bond junction that creates a sequence not contiguous in either parent protein [19, 20]. HIP formation is therefore mechanistically distinct from conventional sidechain PTMs such as oxidation, citrullination, or deamidation. The HIP field includes several complementary levels of evidence [20]. Fusion peptides were first identified as ligands for pathogenic CD4+ T cell clones isolated from NOD islets, and selected HIPs were detected in beta cell material by mass spectrometry [18, 19]. Subsequent studies identified HIP‐reactive T cells in human pancreatic islets and peripheral blood and showed that human HLA‐DQ8 can present insulin‐containing fusion peptides [50]. Mass‐spectrometric analyzes of murine and human islets strengthened evidence that at least some predicted HIPs are naturally formed rather than existing only as synthetic T cell agonists [17].

Enzymatic studies implicate protease‐mediated transpeptidation or reverse proteolysis within the dense, peptide‐rich environment of the insulin secretory granule; cathepsin D can generate several disease‐relevant HIPs, whereas other HIP isomers may arise through different chemical routes [51, 52]. Consequently, HIP formation should not be treated as a single universal reaction. Important uncertainties remain because HIPs may be low‐abundance and isomeric with conventional peptides, while synthetic screens can overestimate physiologically relevant sequences [17]. Activation by a synthetic HIP does not establish sufficient generation and presentation in vivo [19]. The strongest candidates are supported by convergent tissue detection, MHC binding, disease‐associated T cell recognition, and functional activity [51, 52].

### 4.5 Distinguishing PTMs From Other Noncanonical Antigen Sources

Several mechanisms broaden the beta cell repertoire without constituting conventional PTMs. Alternative pre‐mRNA splicing can create new exon junctions or alter isoform abundance; defective ribosomal initiation and translational errors can generate peptides absent from the canonical open reading frame; and altered proteolysis can expose cryptic determinants or change peptide boundaries without creating a fusion junction [12, 17, 18, 19, 51, 53]. These pathways may converge functionally because each can produce peptide–MHC complexes that were absent or poorly represented during tolerance, but they should remain taxonomically separate [12, 18, 19, 20, 27, 28, 51, 52]. Keeping the taxonomy explicit allows apparently similar immune responses to be compared without implying that the antigens were generated through the same biochemical pathway.

## 5 PTMs, MHC‐TCR Recognition, and Epitope Diversification

Modified and noncanonical beta cell peptides become immunologically meaningful only when they are processed, loaded onto susceptible MHC molecules, and recognized by TCRs [15, 22]. Antigenic diversification therefore reflects the interaction of three variables: the changing set of peptides generated by stressed beta cells, the binding preferences and structural flexibility of disease‐associated MHC molecules, and the composition and cross‐reactivity of the available TCR repertoire. This framework helps explain why biochemical modification can be immunologically silent in one individual yet consequential in another, and why apparent broadening of antigen reactivity does not always represent classical epitope spreading [22, 23, 24, 25, 26].

### 5.1 Structural Susceptibility of I‐A^g7^ and HLA‐DQ8

The NOD Class II molecule I‐A^g7^ and the human T1D‐risk molecule HLA‐DQ8 share an important structural feature: both lack the aspartic acid commonly present at position 57 of the MHC Class II beta chain [22, 23]. Loss of this interaction changes the electrostatic and geometric properties of the P9 region and favors peptide repertoires with acidic residues capable of compensating for the missing negative charge [24]. In I‐A^g7^, the altered P9 pocket and the adjacent groove can accommodate diverse peptide registers and binding solutions [21]. This property does not mean that all bound peptides are uniformly unstable or that β57 alone determines disease. Rather, the β57 polymorphism shifts the available peptide repertoire and can permit low‐affinity or unconventional self‐peptide complexes to participate in selection and peripheral activation.

PTMs can exploit this structural susceptibility. Deamidation converts neutral glutamine to negatively charged glutamate and may create a favorable anchor for DQ8 or I‐A^g7^. HIP fusion junctions can also create new anchor combinations that stabilize binding to DQ8 or I‐A^g7^ [40, 46]. Accordingly, increased immunogenicity cannot be inferred solely from improved MHC affinity: a modification may strengthen peptide loading, alter the register, change the exposed TCR‐contact surface, or combine these effects [22]. Structural and functional assays are required to distinguish between them [54].

### 5.2 Register‐Dependent Recognition of Insulin B‐Chain Residues 9–23

Insulin B‐chain residues 9–23 (InsB:9–23) illustrate how peptide register can separate thymic tolerance from peripheral recognition. A Class II peptide can extend beyond the binding groove, but a nine‐residue core occupies the principal anchor positions. Because InsB:9–23 can bind I‐A^g7^ in overlapping registers, different cores from the same peptide expose different residues to the TCR [21]. Studies in NOD mice distinguished one type of T cells, which recognize insulin protein processed by conventional APCs, from another pool of T cells, which respond efficiently to exogenous InsB:9–23 peptide but poorly to intact insulin. The latter recognizes a weakly bound alternative register that is inefficiently generated or displayed during thymic presentation yet is available within pancreatic islets [25].

This register‐shifting model provides a concrete mechanism of thymic escape. T cells are not necessarily ignorant of insulin as a protein; rather, the peptide–MHC configuration encountered in the periphery differs from the dominant configuration that mediates negative selection [25]. Thus, tolerance is imposed against peptide–MHC conformations, not against an abstract antigen name, and stress‐dependent processing or modification can reveal conformations that were poorly represented in the thymus [21, 53]. The same principle may extend beyond insulin. A PTM located at an anchor residue can change which register is preferred, whereas a modification at a solvent‐exposed position may preserve MHC binding but create a new TCR contact [22, 40]. A fusion junction can do both.

### 5.3 Structural Degeneracy and Cross‐Recognition of Beta Cell Peptides

TCR recognition is specific but not absolutely sequence restricted. A single TCR can engage different peptide–MHC complexes when they preserve a sufficiently similar three‐dimensional arrangement of charge, shape, and accessible contact residues [55]. This structural degeneracy is especially relevant in beta cell secretory granules, where highly abundant peptide fragments can be modified or fused into products that differ in primary sequence yet present related surfaces [55, 56, 57].

Structural studies of HIP‐MHC complexes support this principle. An insulin‐IAPP HIP can generate a preferred HLA‐DQ8 binding motif and a distinctive surface for human autoreactive TCR recognition [56]. In the NOD system, I‐A^g7^ displays substantial structural plasticity when bound to an agonistic HIP, and TCR engagement can induce coordinated rearrangements in both the peptide and MHC. In another example, the highly diabetogenic 4.1 TCR recognized seven agonistic HIPs formed from different ChgA and insulin C‐peptide fragments because they shared a common pattern of TCR‐facing residues. This is genuine receptor cross‐reactivity: multiple ligands activate one defined TCR [55, 57].

Cross‐reactivity should not be interpreted as evidence that a cell has acquired additional receptors or that a new clone has been recruited. Nor does recognition of two tetramers necessarily demonstrate equivalent functional avidity, natural presentation of both ligands, or a temporal sequence from one specificity to another. Tetramer binding is influenced by peptide–MHC stability, reagent avidity, TCR density, and activation state [56]. Functional stimulation with each peptide, competition studies, and expression of a cloned TCR in a receptor‐null system provide stronger evidence that one receptor recognizes both ligands [36, 55, 56, 57].

### 5.4 Distinguishing Epitope Spreading From Apparent Diversification

Classical epitope spreading is a population‐level process in which tissue injury and antigen presentation recruit or expand additional lymphocyte clones directed against determinants not dominant in the initiating response [1, 8]. Intramolecular spreading targets new epitopes within the same protein, whereas intermolecular spreading extends to other proteins from the target tissue [7, 10]. A broader panel of recognized peptides is compatible with spreading, but it is not sufficient evidence because the defining observation is the emergence or expansion of additional antigen‐specific clonotypes over time.

Several mechanisms can mimic this pattern of receptor‐level cross‐reactivity [58]. One TCR may cross‐recognize multiple structurally compatible peptide–MHC complexes; a cell may express two TCRα chains; inflammation may activate nearby lymphocytes without new antigen specificity; and trafficking may redistribute pre‐existing clonotypes among blood, lymphoid organs, and pancreas [55, 59]. These alternatives operate at receptor, cell, or tissue levels, whereas classical spreading is a longitudinal population‐level change. The distinction is mechanistically important because broadened tetramer binding or cytokine responses alone do not establish recruitment of new clones [36, 59].

### 5.5 Resolving Diversification

Incomplete allelic exclusion at the TCRα locus allows some T cells to express two α chains, and pairing of an introduced transgenic chain with an endogenous chain can create more than one receptor configuration [60]. A single cell may therefore bind two peptide–MHC reagents because it displays distinct TCRs, not because one receptor is cross‐reactive. This possibility is particularly important in TCR‐transgenic systems that retain endogenous chain rearrangement. Bystander activation does not require newly acquired antigen specificity. Cytokines, costimulatory signals, or local inflammation can activate or expand lymphocytes near an immune response even when their TCRs do not recognize the antigen under study [57, 61].

Similarly, tissue redistribution can alter the apparent frequency or phenotype of antigen‐reactive cells because selective migration into the pancreas, pancreatic lymph node, blood, or spleen may make a response appear or disappear even when the relevant clonotypes have merely moved between compartments. Changes in activation markers, metabolism, or cytokine production therefore cannot establish epitope spreading without antigen‐specific and clonotype‐resolved evidence [60, 61]. A rigorous distinction among these mechanisms requires complementary approaches. Longitudinal peptide–MHC multimer profiling can establish when antigen‐reactive populations appear; paired single‐cell TCR sequencing can determine whether the same or different clonotypes underlie overlapping reactivity; receptor re‐expression can test whether one TCR is sufficient for recognition of multiple complexes; and functional restimulation can distinguish binding from signaling competence [58, 59, 60, 61, 62]. These distinct routes are summarized in Figure 5.

**FIGURE 5:**
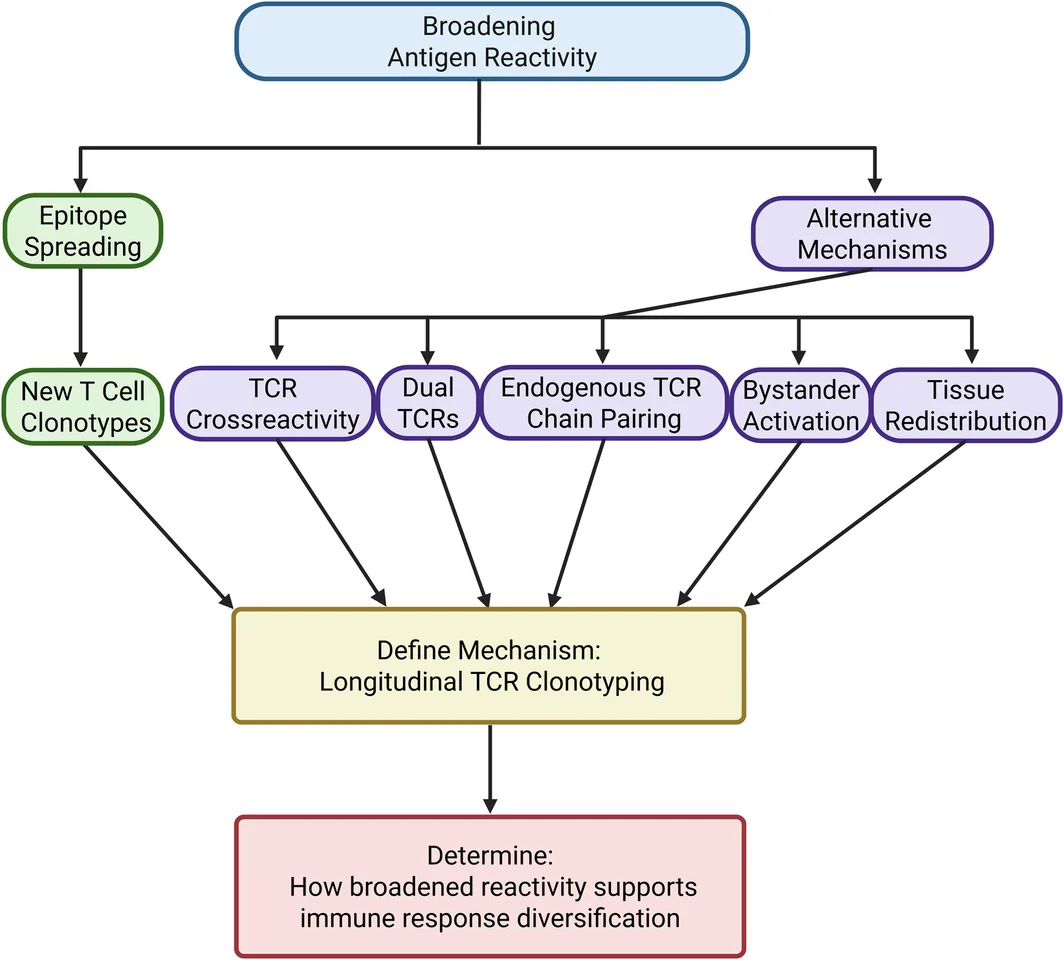
Routes to antigen diversification. Classical epitope spreading recruits or expands clonotypes that recognize new epitopes. Apparent diversification may also result from T cell receptor cross‐reactivity, dual‐receptor expression, bystander activation, or movement of existing clonotypes between tissues. These mechanisms require clonotype‐resolved and functional studies to distinguish them.

## 6 Translational Implications

The expanding repertoire of modified and noncanonical beta cell antigens has implications beyond defining the mechanisms of tolerance loss. If the antigenic landscape changes with beta cell stress and disease progression, biomarkers and therapeutic strategies based exclusively on a static panel of native autoantigens may capture only part of the autoimmune response [12, 13]. Translation of PTM biology will nevertheless require careful distinction between markers associated with disease, antigens that participate directly in tissue injury, and pathways that can be manipulated safely.

### 6.1 Modified‐Antigen Responses as Biomarkers

Conventional islet autoantibodies remain the most established markers for staging presymptomatic T1D, but they do not fully describe the cellular immune response or the changing state of the target tissue [3]. Antibodies and T cells directed against modified antigens may provide complementary information because they can report recognition of structures generated during beta cell stress rather than recognition of the native protein alone. Autoantibodies to oxidatively modified insulin have been detected before diagnosis and, in some cohorts, have improved risk discrimination when added to conventional islet‐autoantibody profiles [45]. Likewise, T cell responses to selected HIPs have been detected in genetically at‐risk individuals before clinical disease [63]. These findings support further evaluation of modified‐antigen responses as indicators of an evolving antigen repertoire.

The most useful PTM‐directed biomarkers may not be universal diagnostic markers. Instead, they may identify biological subgroups, define windows of beta cell stress, or distinguish individuals whose autoimmune responses have progressed from native antigens to stress‐dependent neoepitopes. Longitudinal studies should determine whether certain responses precede seroconversion, emerge during autoantibody expansion, accompany dysglycemia, or primarily reflect established tissue injury [3, 63]. Assay development must also address the low frequency of autoreactive T cells, variation in peptide–MHC multimer performance, HLA restriction, sample handling, and interlaboratory reproducibility [62]. A modified‐antigen assay should demonstrate added value beyond age, genetics, conventional autoantibodies, metabolic measures, and other biomarkers before it is considered clinically informative.

### 6.2 Implications for Antigen‐Specific Tolerance

Antigen‐specific therapies seek to suppress pathogenic immunity while preserving broad immune competence [2]. The evolving neoepitope model complicates this goal because tolerance induced to a single native determinant may not control lymphocytes recognizing modified versions of that antigen, fusion peptides, or epitopes derived from other beta cell proteins [12]. A single‐antigen strategy may be most effective early, before extensive diversification, whereas later disease may require multiepitope formulations or combinations selected for the antigenic repertoire present at that stage [1]. This does not mean that every detectable epitope should be incorporated into a tolerogenic product.

Some responses may be cross‐reactive manifestations of the same clonotype, secondary consequences of tissue injury, or immunological markers without pathogenic function [12]. Rational antigen selection should prioritize epitopes that are naturally presented, associated with disease‐relevant HLA molecules, recognized by pathogenic or inflammatory lymphocytes, and detectable during the intended treatment window. Native and modified versions of an antigen may need to be considered together when one TCR can recognize both [55]. Conversely, evidence of classical epitope spreading may justify targeting multiple independent clonotypes [1]. Longitudinal antigen profiling could therefore support stage‐adapted therapy rather than a single formulation applied uniformly throughout disease.

### 6.3 Combining Immune Modulation With Tissue‐Directed Intervention

The stress‐to‐neoepitope model also identifies the beta cell as a therapeutic target. Reducing ER or redox stress could improve beta cell function while decreasing the formation, release, or presentation of modified antigens [13, 32]. In mouse models, manipulation of the unfolded protein response or treatment with chemical chaperones has reduced beta cell stress and altered autoimmune‐diabetes progression, providing proof of concept that tissue state can influence immune susceptibility. Antioxidant or redox‐modulating approaches may similarly limit oxidative PTMs, although broad suppression of physiological redox signaling could impair insulin secretion and normal cellular defense [38, 64].

TGM2 or PAD inhibition offers a more direct strategy for reducing selected deamidated, cross‐linked, or citrullinated products [39, 40, 42]. However, these enzymes participate in processes beyond neoepitope formation, including extracellular‐matrix regulation, wound responses, cell signaling, chromatin regulation, and innate immune functions. Systemic or sustained inhibition could therefore produce effects unrelated to beta cell antigenicity. Tissue‐directed therapy is most likely to complement, rather than replace, immune modulation because reducing antigen generation may not eliminate memory T cells or clones already recruited through epitope spreading.

### 6.4 Patient Stratification and Stage‐Adapted Intervention

The biological relevance of a modified epitope is determined partly by the patient in whom it arises. HLA genotype governs which peptides can be presented, while TCR repertoire, age, inflammatory history, beta cell reserve, and disease stage influence whether presentation produces a measurable or pathogenic response [22]. A deamidated peptide favored by HLA‐DQ8, for example, may have limited relevance in an individual whose HLA molecules do not present that sequence efficiently [46]. Future stratification strategies should therefore integrate HLA context, conventional autoantibodies, metabolic status, markers of beta cell stress or death, and the individual PTM‐reactive lymphocyte repertoire [3, 12, 13]. This approach could identify who is most likely to benefit from a particular antigen‐specific or tissue‐directed therapy and when it should be administered [63]. Ultimately, the translational value of PTM biology lies not in replacing established T1D markers or immune interventions, but in explaining heterogeneity that those approaches do not capture and enabling combinations tailored to the evolving interaction between beta cell state and autoreactive immunity.

## 7 Conclusions

Beta cell antigenicity is not a fixed property defined solely by the expression of native autoantigens. Instead, the antigenic landscape changes with beta cell physiology, inflammatory exposure, and disease progression [13]. The high secretory demand of the beta cell creates an intrinsic vulnerability to ER and redox stress, while cytokines, viral signals, calcium dysregulation, and immune‐mediated injury can further alter protein folding, processing, and modification [29, 32, 38]. These conditions generate modified and noncanonical peptide structures that may have been absent or poorly represented during central tolerance. In genetically susceptible individuals, particularly those expressing disease‐associated HLA molecules, these newly available peptide–MHC complexes can create additional opportunities for autoreactive lymphocyte recognition.

The compiled evidence provides an important mechanistic bridge between beta cell stress and altered antigen recognition [13, 15]. This body of work helped advance the field from a static catalog of beta cell autoantigens toward a model in which the state of the target tissue actively shapes the immune repertoire [29, 32]. Within this model, stress‐dependent antigen generation can diversify the peptide repertoire available for presentation, while epitope spreading, TCR cross‐reactivity, dual‐TCR expression, and clonal recruitment provide distinct routes through which immune recognition may appear to broaden [1, 55]. Resolving these mechanisms will require longitudinal, tissue‐resolved, and clonotype‐resolved studies that establish when modified antigens arise, whether they are naturally presented, and which responses contribute directly to beta cell injury [12, 13]. Ultimately, recognizing beta cell antigenicity as an evolving process may improve biomarker development, patient stratification, and therapeutic strategies that combine immune tolerance with preservation of beta cell homeostasis.

## Funding

This review was funded by the National Institute of Diabetes and Digestive and Kidney Diseases (NIDDK) under project 7R01DK132583 and by Breakthrough T1D (BT1D) through a Strategic Research Agreement (SRA), Project 1‐PNF‐2025‐1642‐A‐N, awarded to Dr. Jon D. Piganelli. This study is also supported by an NIH R21 award (1R21AI193399) to Jon D. Piganelli.

## Conflicts of Interest

The authors declare no conflicts of interest.

## Data Availability

Data sharing is not applicable to this article because no new datasets were generated or analyzed in this review.
